# Effect of Melt-Derived Bioactive Glass Particles on the Properties of Chitosan Scaffolds

**DOI:** 10.3390/jfb10030038

**Published:** 2019-08-13

**Authors:** Hamasa Faqhiri, Markus Hannula, Minna Kellomäki, Maria Teresa Calejo, Jonathan Massera

**Affiliations:** Faculty of Medicine and Health Technology, Tampere University, 33720 Tampere, Finland

**Keywords:** bioactive glass, chitosan, composites, bone tissue engineering

## Abstract

This study reports on the processing of three-dimensional (3D) chitosan/bioactive glass composite scaffolds. On the one hand, chitosan, as a natural polymer, has suitable properties for tissue engineering applications but lacks bioactivity. On the other hand, bioactive glasses are known to be bioactive and to promote a higher level of bone formation than any other biomaterial type. However, bioactive glasses are hard, brittle, and cannot be shaped easily. Therefore, in the past years, researchers have focused on the processing of new composites. Difficulties in reaching composite materials made of polymer (synthetic or natural) and bioactive glass include: (i) The high glass density, often resulting in glass segregation, and (ii) the fast bioactive glass reaction when exposed to moisture, leading to changes in the glass reactivity and/or change in the polymeric matrix. Samples were prepared with 5, 15, and 30 wt% of bioactive glass S53P4 (BonAlive ^®^), as confirmed using thermogravimetric analysis. MicrO–Computed tomography and optical microscopy revealed a flaky structure with porosity over 80%. The pore size decreased when increasing the glass content up to 15 wt%, but increased back when the glass content was 30 wt%. Similarly, the mechanical properties (in compression) of the scaffolds increased for glass content up to 15%, but decreased at higher loading. Ions released from the scaffolds were found to lead to precipitation of a calcium phosphate reactive layer at the scaffold surface. This is a first indication of the potential bioactivity of these materials. Overall, chitosan/bioactive glass composite scaffolds were successfully produced with pore size, machinability, and ability to promote a calcium phosphate layer, showing promise for bone tissue engineering and the mechanical properties can justify their use in non-load bearing applications.

## 1. Introduction

The S53P4 bioactive glass has a nominal composition (in wt%) of 53% SiO_2_, 4% P_2_O_5_, 23% Na_2_O, and 20% CaO, and has been cleared for clinical use by the Food and Drug Administration (FDA) [1,2]. As reported by van Gestel at al., the use of S53P4 is increasing in bone graft applications due to its ability to facilitate and stimulate bone formation and bone defect healing [3]. However, the use of this bioactive glass is hindered by a few limitations. Studies by Lindfors et al. on the implantation of large bioactive glass particles (1–4 mm) revealed that some material remained undissolved at the surgical site even at 14 years post-surgery [4]. This indicates that when large glass particles are used in bone surgery, the small surface area leads to incomplete glass dissolution and encapsulation of the glass particles within the newly formed bone. Furthermore, while three-dimensional (3D) porous constructs would be optimal for bone reconstruction and vascularization, typical bioactive glasses are mainly used as granules. The reason lies in the highly disrupted glass structure that leads to rapid and uncontrolled crystallization [5]. Even more, crystallization of bioactive glasses during sintering and its impact on bioactivity are still under debate [6].

For many years, chitosan has been an attractive polymer in bone tissue engineering and regenerative medicine. Chitosan is a naturally occurring polymer which has been found to promote attachment and proliferation of osteoblasts, as well as formation of a mineralized bone matrix, while evoking minimal foreign-body response [7]. Furthermore, while only a few compounds, i.e., hydroxyapatite (HA) and bioactive glass, are classified as bioactive, bioresorbable, and osteoconductive, chitosan is one natural polymer which has demonstrated osteoconductivity when modified with covalently-bonded imidazole groups [8].

Chitosan has rapidly become a material of choice in the development of biocomposites for bone tissue engineering. Typically, chitosan is mixed with synthetic hydroxyapatite and/or tricalcium phosphate. Various processes developed to produce the scaffolds have been reported in [9]. Over time, studies on chitosan-ceramic biocomposites have emerged, demonstrating that the combination of a natural polymer with an osteoconductive ceramic holds promise for bone tissue engineering [9]. More recently, the addition of nano-bioactive glass particles has been investigated as a means to endow the biomaterials with osteoinductive properties. Correia et al. have developed a chitosan/bioactive glass nanoparticle scaffold with shape memory properties [10]. As expected, the authors demonstrated that the addition of the filler increased the stiffness of the materials and promoted the formation of HA within seven days. In most studies, glass particle size is found in the range of 10 nm–5 µm [11,12]. However, using small particles implies fast particle degradation, rapid ion leaching, and unsustained ion release.

In this study, chitosan/bioactive glass composites were prepared and characterized as potential biomaterials in bone tissue engineering. The melt-derived glass particles used in this study were sieved to <50 µm, leading to particles in the size range of 1–55 µm and average size of 28 ± 7 µm. The use of melt-derived glass particles is of particular interest since it reduces the risk of fast glass dissolution, provides a sustained ion release, while enabling one to have higher ability to tune the glass compositions based on the aimed application. Here, as proof of concept, the FDA-approved S53P4 bioactive glass was used. Chitosan was loaded with 0, 5, 10, and 30 wt% of bioactive glass as measured by thermogravimetry. The scaffold porosity was defined by micrO–Computed tomography (µCT). In vitro dissolution was conducted in TRIS buffer solution to evidence the ion release kinetics. Ion release was quantified using ICP-OES. Change in structure upon material immersion was assessed by FTIR-ATR. Finally, the mechanical properties of the composite were measured in compression.

## 2. Materials and Methods

### 2.1. Glass processing 

The silicate glass, S53P4 (53.86SiO_2_-22.66Na_2_O-21.77CaO-1.72P_2_O_5_ in mol%), was produced by traditional melt quenching. A mixture of sand (99.4% pure SiO_2_) and analytical grade Na_2_CO_3_, CaCO_3_, and CaHPO_4_.2H_2_0 was melted in a platinum crucible at 1400 °C for 3 h. The melt was then casted into a graphite mold and annealed at 40 °C below the glass transition temperature to remove any residual stress. The ingots were crushed and sieved to a particle size <50 µm.

### 2.2. Chitosan/glass composites 

2 wt% of Chitosan (Acros Organics) with molecular weight 100,000–300,000 g/mol was dissolved in 1% acetic acid as reported in [13]. The solution was stirred using a magnetic stirrer (RO5, IKA^®^-Werke GmbH &Co, Staufen, Germany) until complete chitosan dissolution. The viscous solution was left to stir overnight at 30 rpm to set and prevent bubble formation.

Composite scaffolds were prepared by slowly adding the desired amount of bioactive glass (particles <50 µm) under stirring. The glass was added slowly to prevent particle aggregation. The pure chitosan solution (used as control) was labeled as 0 wt% and the composites were labeled based on their glass content. The viscous solutions were then poured into 30 mL test tubes, frozen overnight, and finally freeze-dried at −100 to −110 °C for 48 h (Heto Drywinner CT/DW 110 freeze-dryer Jouan Nordic, Allerød, Denmark).

As suggested in [13], samples were thereafter neutralized using 0.2 M NaOH. Test tubes were filled with the neutralizing solution and left at room temperature for 30 min. Samples were then repeatedly washed with distilled water until reaching neutral pH. Samples were thereafter frozen overnight and freeze-dried again for 24 h. The obtained 3D scaffolds were cut into discs (5 mm thick) using a scalpel and maintained in a desiccator until further use.

### 2.3. Thermal analysis

An STA 449 F1 Jupiter-combined thermogravimetric analysis (TGA)/differential thermal analysis (DTA) was employed to assess the glass content in the chitosan matrix. All measurements were performed at least three times per sample. Samples were introduced in an Al_2_O_3_ pan and the temperature was increased to 1200 °C at 10 °C/min under a flow of N_2_. The measurements were performed on six samples per composition.

### 2.4. Porosity

The porosity was analyzed using X-ray micrO–Computed tomography (µCT). MicroXCT-400 (Carl Zeiss X-ray Microscopy, Inc., Pleasanton, CA, USA) was used with tube voltage 140 kV and current 71 µA. Pixel size was 5.6 µm. Porosity analysis was done with Fiji [14] using a BoneJ [15] plugin. µCT visualizations were done with Avizo 9.1 (FEI Visualization Sciences Group, Berlin, Germany). Porosity, pore size, and pore size distribution were quantified on one representative sample over a volume of 4 × 4 × 3 mm^3^.

### 2.5. Mechanical properties

The mechanical properties of the chitosan scaffold and composites were investigated by compression using an Instron ElectroPluse E1000 (High Wycombe, UK). To calculate the maximum strength and the Young’s modulus, the height and diameter of the samples were measured using a Caliper. To compensate for the uneven surface, samples were pre-compressed to 50% strain. Four parallel measurements were carried out for each sample.

### 2.6. Structural properties

Fourier Transform Infrared Spectroscopy (Spectrum One FT-IR spectrometer, Perkin Elmer, Waltham, MA, USA) was used to assess the samples’ chemical structure. The system was used in attenuated total reflectance (ATR) mode. The crystal used was a diamond, the resolution was 4 cm^−1^, and eight scans were accumulated within the 650–4000 cm^−1^ range. All spectra were baseline-corrected and normalized to the band with maximum intensity for ease of absorption bands’ intensity comparison.

### 2.7. In vitro dissolution

Both the composites and the neat chitosan scaffold were immersed in 0.05 M TRIS buffer solution, pH = 7.40 ± 0.02, and T = 37 °C, for up to 5 weeks in a shaking incubator (100 rpm). The mass of each sample was measured before immersion. The volume of TRIS solution was adjusted to maintain a constant surface area to volume ratio as suggested in [16]. At each time point, the pH of the solution was measured and 1 mL of the solution was collected and diluted 10 times with distilled water for ion concentration analysis using an ICP-OES (ICP-OES 5110, Agilent technology). The elements studied and the wavelength analyzed by ICP were P (253.561 nm), Si (250.690 nm), Ca (422.673 nm), and Na (568.821 nm).

The mass loss and water uptake were also assessed. The water uptake was calculated using Equation (1) [16]:

(1) $$water\;uptake\left(\%\right)=100\times \frac{m_{1}-m_{2}}{m_{2}}$$

where *m*_1_ is the wet mass measured after immersion in TRIS, and *m*_2_ is the dry mass of the samples post immersion.

Similarly, the mass loss was measured using Equation (2):

(2) $$mass\;loss\left(\%\right)=100\times \frac{m_{0}-m_{2}}{m_{2}}$$

where *m*_0_ is the initial mass of the sample before immersion.

The chemical structure of the samples was measured post-immersion using FTIR, using the same procedure described above.

## 3. Results and Discussion

**Figure 1:**
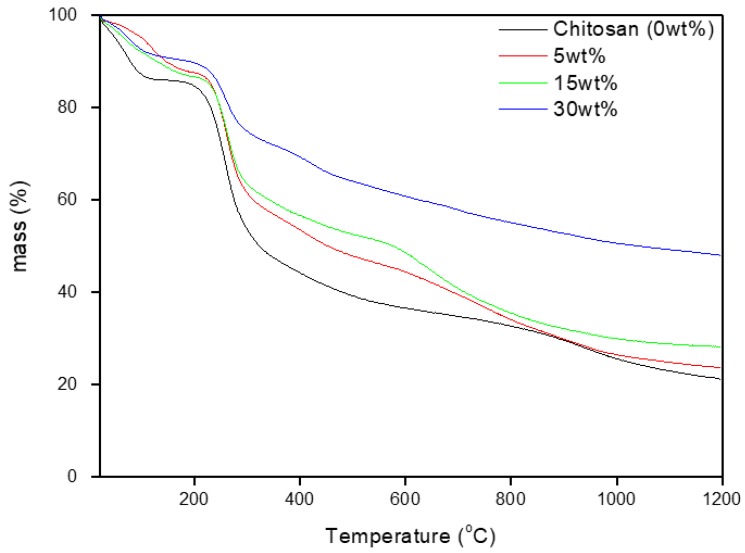
TGA thermogram of the samples of investigation.

Figure 1 presents the TGA thermogram of the neat chitosan scaffold and the composites loaded with bioactive glass particles (particle size <50 µm). The thermogram was recorded up to 1200 °C. Only 20 ± 3% of the original mass of the neat chitosan scaffold was left after the heating cycle, which accounted for the char remaining in the alumina crucible. The residual mass measured here is in good agreement with previously reported TGA data [17,18]. With increasing glass content, one can see that the percentage of residual mass increases. From the TGA thermogram and keeping in mind that 20% of the neat chitosan mass was left after the heating cycle, one can back-calculate the glass content in the composites using the equation below:

(3) glass(%)=Residualcompositemass(%)−Residualchitosan mass(%)0.8

where the “*Residual composite mass (%)*” is extracted from the TGA (at 1200 °C) and the “*Residual chitosan mass (%)*” is 20% as evidenced on the TGA thermogram of the chitosan alone. Using the equation above, one can calculate that the glass content in the three composites is, respectively, 5 ± 3, 11 ± 5, and 36 ± 12 wt%. The larger standard deviation observed for the sample with the highest glass content can be explained by the higher tendency of concentrated samples to undergo particle agglomeration and segregation, in an effect that is further enhanced by the large size of the particles used in this study.

The TGA thermogram of the chitosan scaffold shows the typical mass loss as reported in previous studies [19,20,21]. The mass loss in the 40–175 °C range (~14%) can be attributed to the loss of physically-absorbed and loosely-bonded water molecules. The steepest decrease in mass, in the 200–400 °C range, can be ascribed to further dehydration, deacetylation, and depolymerization of chitosan. The mass loss does not reach a plateau but rather continues to decrease at temperatures higher than 400 °C, due to residual decomposition. It is important to note that while chitosan thermal decomposition is well documented in the literature, this is, to the best of our knowledge, the first study where the thermal degradation of chitosan has been investigated at temperatures higher than 800 °C. Therefore, the last decrease in mass, at temperatures above 800 °C, is speculated to be due to the evaporation of volatile compounds in the chitosan char. With the addition of the bioactive glass, the mass losses related to dehydration, deacetylation, and depolymerization are shifted to higher temperatures. However, the residual decomposition and evaporation of volatile by-products from the chitosan burn-off occur at lower temperatures than for the neat chitosan. This is attributed to the lower chitosan content in the composites, but may also arise from small structural differences in the polysaccharide itself, due to interaction between the polymer and the glass surface, favoring the thermal degradation of chitosan.

**Figure 2:**
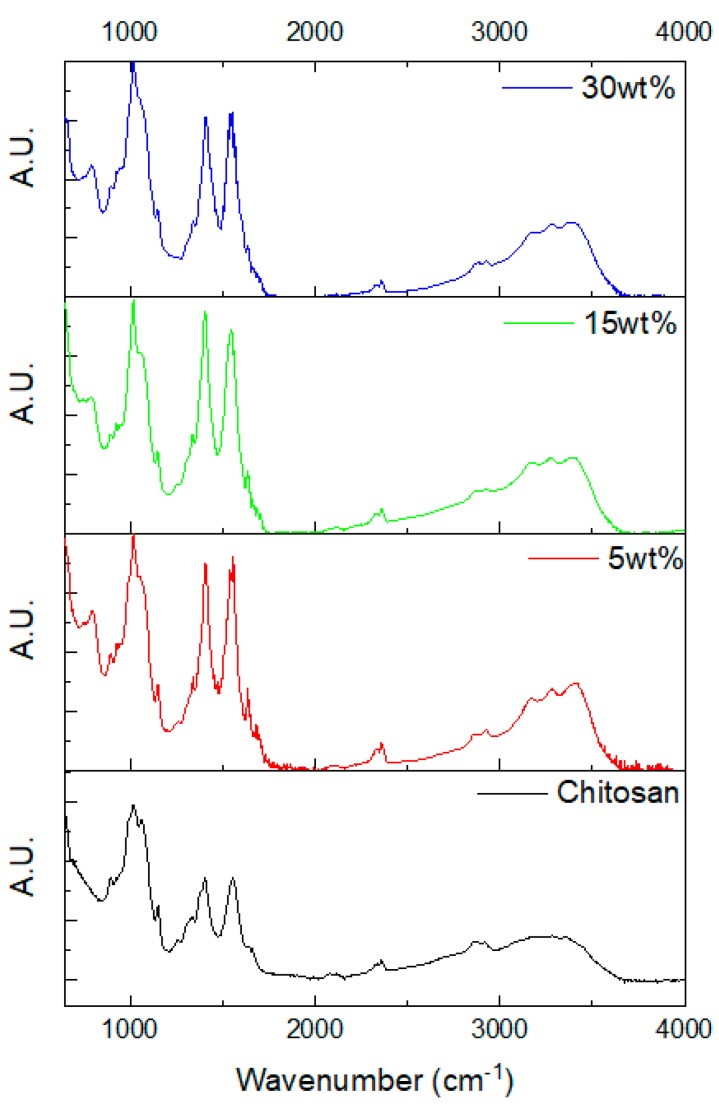
FTIR spectra of chitosan and the composites of investigation.

The structural properties of the scaffolds were studied by ATR-FTIR. The spectra are presented in Figure 2.

The full absorption band attribution is reported in Table 1 for chitosan based on [19,21,22,23,24]. A broad band in the 3000–3500 cm^−1^ region is present in the FTIR spectrum of chitosan, related to the vibration of water molecules. This broad band becomes a triplet with the incorporation of the bioactive glass, attributed to water in silicate glass as reported in [25]. The addition of glasses also leads to the appearance of a new peak in the 793–800 and 926 cm^−1^ region related to Si–O–Si and Si–O vibration in silicate glasses, respectively [22,26]. The absorption band at 893 cm^−1^, attributed to the saccharide structure, decreases in intensity with the presence of bioactive glass. The shoulders at the left- and right-hand side of the main absorption band (at 1015 cm^−1^ and attributed to C–O–C and C–O vibration) decreased in intensity in the composites’ spectra. The absorption band at 1409 (C–O and CO_3_ vibrations) and 1552 cm^−1^ (amide II) increased in intensity with the glass incorporation due to the interaction between the amide groups and the glass surface [23]. Finally, with the presence of bioactive glass in the chitosan matrix, a peak at 1638 cm^−1^ appeared. This was assigned to binding of the chitosan to the bioactive glass as reported by Maji et al. [12]. Overall, the presence of the glass in the chitosan structure is evidenced by ATR-FTIR. Evidence of the interaction between the chitosan and the bioactive glass was revealed, as shown by the change in the chitosan structure.

**Figure 3:**
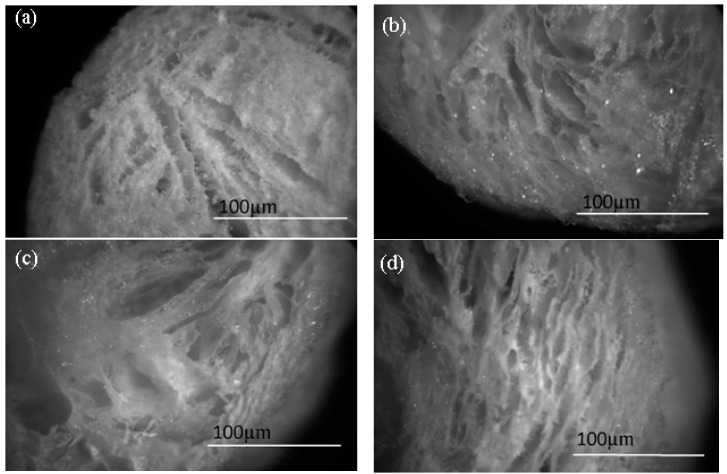
Optical microscopy images of the samples of investigation (chitosan (**a**)) and composites with 5 wt% (**b**), 15 wt% (**c**), and 30 wt% (**d**).

The microstructure of the samples was analyzed by optical microscopy. Optical microscopy images of the four produced samples can be seen in Figure 3 and exhibit a flaky structure.

**Figure 4:**
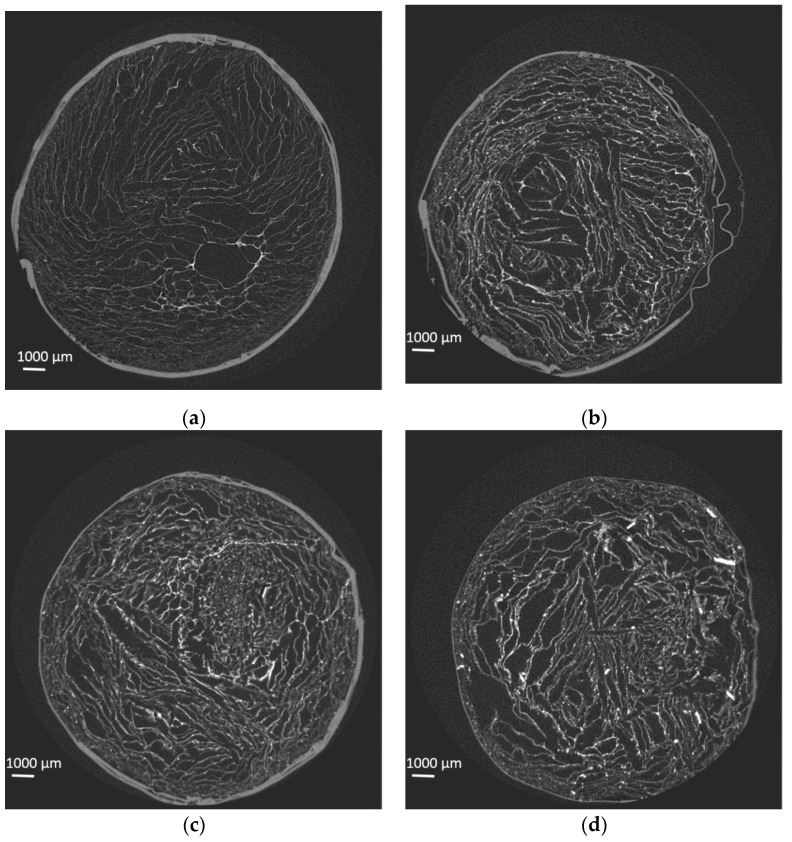

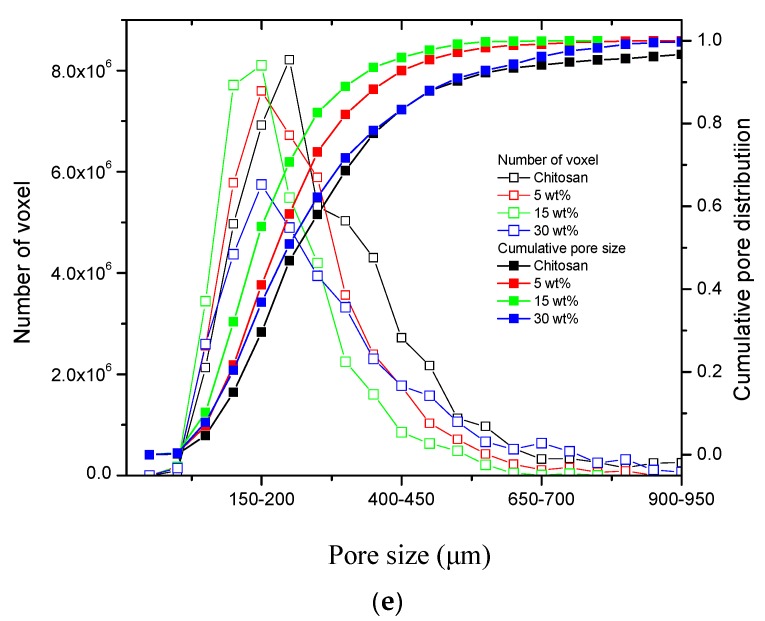
µCT section of the chitosan scaffold (**a**), as well as of samples prepared with the targeted 5 wt% (**b**), 15 wt% (**c**), and 30 wt% (**d**) of bioactive glass particles. (**e**) The pore size and cumulative pore distribution in the scaffolds.

The porosity of the samples was assessed by µCT. Figure 4 shows the µCT section of the chitosan scaffold (**a**) as well as of samples prepared with the targeted 5 wt% (**b**), 15 wt% (**c**), and 30 wt% (**d**) of bioactive glass particles. Figure 4e presents the pore size distribution, as well as the cumulative pore fraction in the samples.

All materials were found to be highly porous, with the highest porosity being found for the neat chitosan scaffold (88%). Interestingly, while a decrease in the overall porosity was generally seen with the addition of bioactive glass, the presence of high amounts of glass was found to reverse this trend. Indeed, the porosity of the composites containing 30 wt% of glass particles was higher (75%) than the porosity of the composites containing only 15 wt% of glass (66%), which were, in fact, the least porous samples of all. The composites containing 5 wt% of glass particles had a similar porosity to the 30 wt% containing scaffolds (75%). The µCT images of the composites containing 5 wt% of bioactive glass exhibited bright dots within the chitosan struts. The density of bright dots increased with increasing glass particle loading, suggesting that those dots are indeed the glass particles within the natural polymer matrix. An increase in the glass particles up to 15 wt% seems to lead to homogeneous dispersion of the glass particles. At the highest loading, a clear presence of particle aggregates can be seen in the image (Figure 4d). The pore size distribution presented in Figure 4e exhibits a shift toward smaller pore size and a narrower pore size distribution when the glass loading increases from 0 to 15 wt%. This is in agreement with the observations made for other composites using different foaming techniques [27]. At the highest glass loading (30 wt%), the pore size distribution broadens again and the median pore size shifts back to similar values than for the chitosan alone. This can be attributed to the particles’ agglomeration due to segregation of glass particles prior to freeze drying.

**Figure 5:**
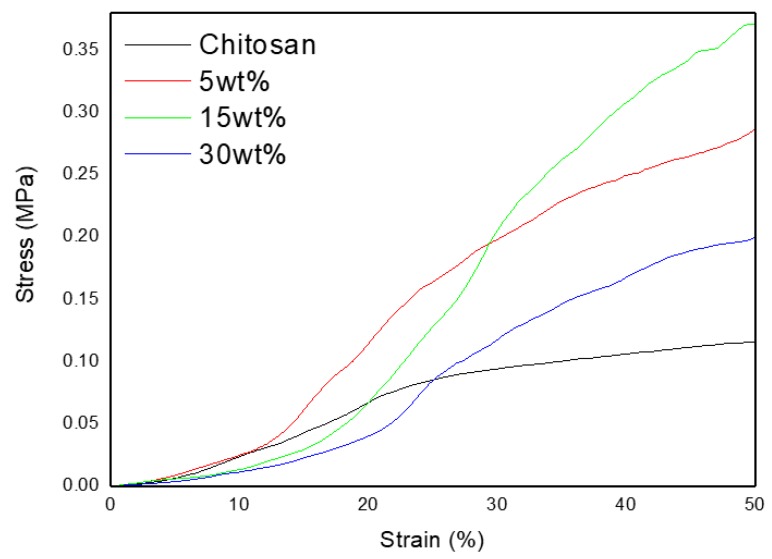
Representative stress vs. strain curves, upon compression test of the materials of investigation.

The mechanical properties of the chitosan and the composites were recorded in compression, and the stress-strain curves are reported in Figure 5.

The tensile strength of the neat chitosan scaffold was estimated as 0.12 ± 0.06 MPa at 50% deformation, and the Young’s modulus was 0.23 ± 0.05 MPa. These values are in good agreement with data reported by other authors on similar porous chitosan scaffolds [28]. When the glass content was increased from 0 to 15 wt%, an increase in the tensile strength (up to 0.4 ± 0.1 MPa) and Young’s modulus (up to 0.78 ± 0.01 MPa) was recorded. An increase in the mechanical properties could be assigned to an increased interaction between the hydrated layer of the bioactive glass and/or by ionic interaction from cationic ions leaching from the glass during the composites processing [29]. While a further increase was expected at the highest glass content, the mechanical properties of the 30 wt% composite were lower than that of the other composites, and only slightly higher than the neat chitosan scaffold. Similar behavior for the elastic modulus was reported for chitosan/nanohydroxyapatite (nHA) composite membranes [30]. The maximum in the elastic modulus was reported at 20% loading. However, in [30], an almost linear decrease in the tensile strength was reported as a function of nHA loading. In that work, as in ours, this decrease in mechanical properties is most likely due to the agglomeration of glass particles in the scaffold, as seen in our µCT images, leading to an inhomogeneous composite structure. While the mechanical properties remained too low for application in bone structure, those scaffolds could be used in addition to more robust 3D bioceramic scaffolds. Furthermore, such biomaterials may find application in wound healing. Finally, it is interesting to note that, in the past, 3D collagen scaffolds with low mechanical properties were investigated to understand the mechanobiology of cancer cells [31].

**Figure 6:**
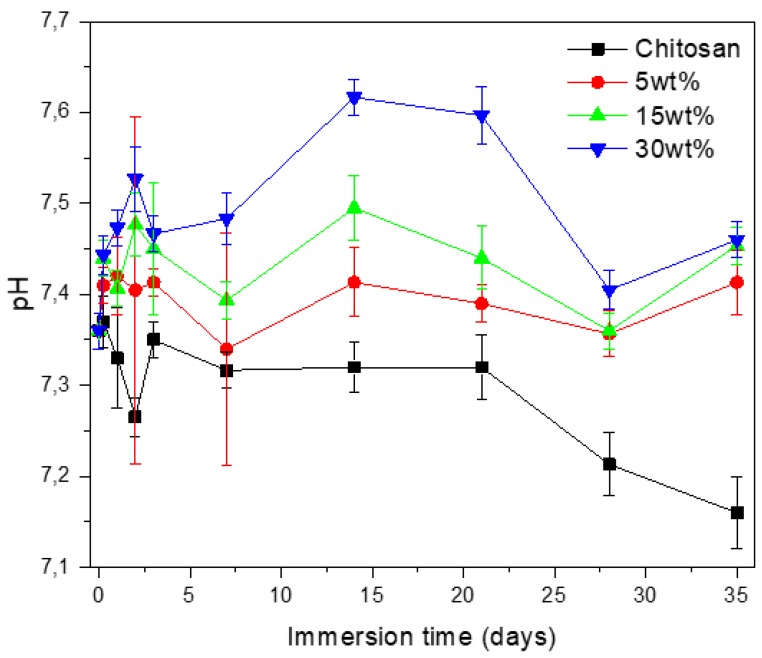
pH of the immersion solution as a function of immersion time (data are the average of six measurements, and error bars correspond to the standard deviation. If the standard deviation was lower than 0.02 then the error of measurement of the constructor was used).

The measured physicochemical properties are summarized in Table 2:

The in vitro dissolution of the chitosan scaffolds and composites was assessed by monitoring pH changes over an incubation period of 35 days (Figure 6).

When immersing the neat chitosan scaffold in TRIS buffer, the pH remained mostly unchanged during 20 days, and then decreased at longer immersion times. In vitro chitosan degradation typically starts with the random splitting of the β-glycosidic bonds, followed by deacetylation [32]. In our work, the decrease in pH marked the onset of chitosan deacetylation and the associated release of acetic acid. Upon immersion of the composite containing 5 wt% of bioactive glass, the pH remained constant throughout the dissolution study, whereas upon immersion of the composites with higher bioactive glass content, an initial increase in the pH was observed, followed by a drop at around 20 days of immersion. These results suggest that the release of alkaline and alkaline-earth elements from the glass particles leads to a buffering effect [33]. The pH decrease induced by chitosan is compensated by the increase in pH resulting from the dissolution of bioactive glass [34,35]. The average water absorption was ~1200 ± 250% for all materials tested, regardless of the glass content, after 6 h of immersion and remained constant over the all immersion study. At the end of the immersion test, the mass loss was ~12 ± 2% for the neat chitosan and 23 ± 4% for all composites, independent of the glass content.

**Figure 7:**
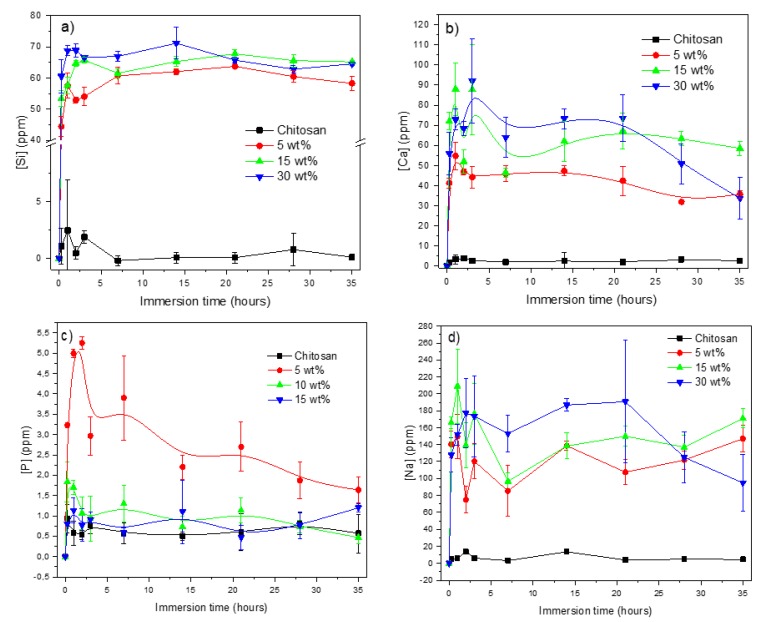
Release profile of Si (**a**), Ca (**b**), P (**c**) and Na (**d**) ions in TRIS buffer solution as a function of immersion time.

For all composites, the release of ions seems to occur mainly at early immersion times (up to 72 h) and then it levels off (Figure 7). An increase in the bioactive glass content leads, as expected, to higher ion release. The only exception concerns the release of P (Figure 7c), which exhibits significantly higher values for the composites containing the lower amount of bioactive glass. FTIR analysis was conducted on all immersed samples to investigate changes in the chemical structure of the scaffolds upon dissolution.

**Figure 8:**
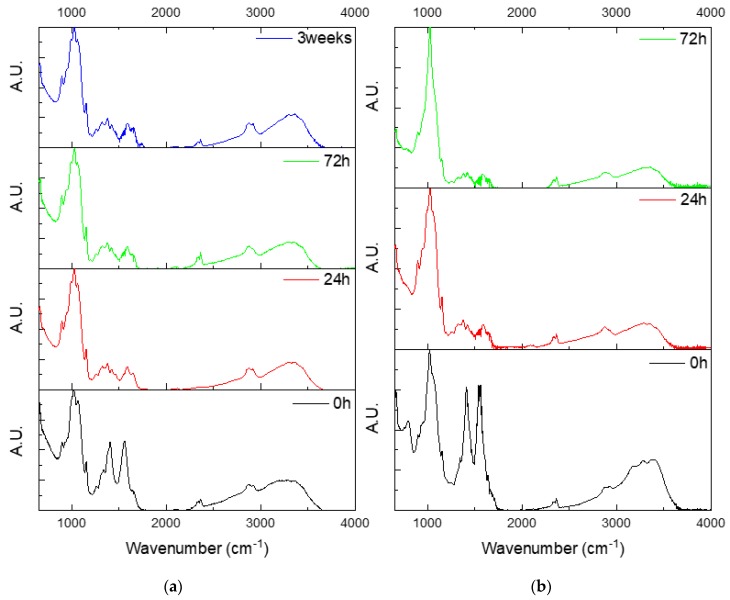
FTIR spectra of (**a**) the chitosan scaffolds upon immersion for up to 3 weeks, and (**b**) composites containing 15 wt% of bioactive glass.

Figure 8a presents the FTIR spectra of the chitosan scaffolds upon immersion for up to 3 weeks. After 24 h of immersion, a strong decrease in the bands located at 1401 (C–O bonds) and 1551 cm^−1^ (N–H bonds in Amide II) was observed. It is known that chitosan dissolution through hydrolysis is slow compared to enzymatic degradation. This is even more significant if the degree of crystallinity is high. In Figure 8a, one can see that structural modification occurs rapidly (up to 24 h), and thereafter, no further structural changes occur, indicating that the polymer remains stable over the dissolution test. The FTIR spectra of the composites containing 5 wt% of bioactive glass (data not shown) was similar to the pure chitosan. However, composites containing 15 wt% of bioactive glass (Figure 8b) experienced significant structural modifications during the immersion period. As discussed previously, the decrease in the intensity of the peaks at 1401 and 1551 cm^−1^ suggests that N–H and C–O bonds are broken during the initial 24 h of immersion. In addition, the FTIR spectrum shown in Figure 8b also discloses the disappearance (at 24 h of immersion) of the band at 788 cm^−1^, attributed to the silica network of the bioactive glass. This confirms the dissolution of the glass particles at short immersion times. More interestingly, the main band changes in shape and position to reveal a main peak at 1024 cm^−1^, and an additional peak at 894 cm^−1^ and a shoulder at 962 cm^−1^. The shape and position of the peaks are highly similar to the FTIR trace of carbonated HA reported in the literature, upon dissolution of bioactive glass [36,37].

## 4. Conclusions

In this study, the processing and characterization of chitosan/melt-derived bioactive glass S53P4 is reported. The scaffolds were obtained by thoroughly mixing the chitosan and the bioactive glass, followed by freeze drying. As expected, the presence of bioactive glass leads to ion leaching, which is able to supersaturate the immersion solution, in turn inducing precipitation of reactive layer. This reactive layer gives a first indication that the developed materials are promising from a bioactive point of view. The produced scaffolds all have a porosity (pore size >100 µm and porosity >60%) suitable for bone tissue engineering. However, the mechanical properties of the neat chitosan could only be slightly increased by the presence of the secondary phase. This was most likely due to interaction with the glass hydrated layer and/or ionic interaction with ions leached from the glass during the process leading to a higher cross linking of the chitosan. It is also important to point out that enhanced mechanical properties were not achieved for the highest loading. Indeed, the scaffolds containing the higher content of bioactive glass clearly showed signs of agglomeration of glass particles, therefore increasing inhomogeneities in the composites structure and properties.

Overall, the developed materials are promising from the regeneration of hard tissue perspective. The use of melt-derived glasses opens the path to greater possibilities to tailor the inorganic phase composition in order to match the intended application (fast vs. slow glass dissolution, presence of therapeutic ion if required). While here the mechanical properties were only slightly improved, these developed materials can be used in conjunction with other, more mechanically stable bioceramic scaffolds. A path that should be investigated is the use of a crosslinker between the chitosan and the bioactive glass particles in order to prepare and hybrid biomaterials with enhanced mechanical/rheological properties. Finally, while these materials are primarily intended as bone substitutes, a large amount of literature is available for use of chitosan and bioactive glass in wound healing, where the mechanical properties are not of the utmost importance.

## Figures and Tables

**Table 1:** FTIR absorption band attribution.

Wavenumber (cm^−1^)	Identification of Absorption Bond
3496–3440	OH group
1377	O–H and N–H axial stretching, NH group-stretching vibration
3345, 3370	O–H band that overlaps N–H band, –NH_2_ and –OH groups
2910, 2913, 2859	C–H stretching
1300, 2926, 2880, 665	C–H bending vibration
1421, 1322	OH, CH vibration in the ring
2877,1421, 1322, 1249	CH_2_ in pyranose ring
1422	Vibration of C–OH group
1724, 1580, 1395	C=O, Stretching vibration C=O
1646, 1642	C=O in amide I group
1653, 1657	Amide I
1650	Stretching vibration of amide I
1381	CH_3_ in amide group
1096, 1030	C–O group in amide group
1580	Amide II
1562, 1552	Amide II band due to N–H bending (Amide II)
1320	Amide III
1320, 1590	Amino characteristic peaks
1593	NH_2_ bending vibration in amino group
1417	Coupling C–N axial stretching
1249, 1075, 1033	C–O group, C–O vibration stretching
1152, 1153	–C–O–C– bridge
1153–897	Polysaccharide, C–O and C–O–C
1085	C–O–C bond
1065, 1150, 1024	C–O–C symmetric, C–O–C asymmetric vibration
1380	Stretching vibration of methyl group
893, 1153	Saccharide structure

**Table 2:** Physicochemical properties of the investigated samples.

Sample Name	Measured Inorganic Mass (wt%)	Porosity	Tensile Strength at 50% Deformation (MPa)	Young’s Modulus (MPa)
Chitosan (0 wt%)		88%	0.12 ± 0.06	0.23 ± 0.05
5 wt%	5 ± 3	75%	0.4 ± 0.1	0.78 ± 0.01
15 wt%	11 ± 5	66%	0.29 ± 0.05	0.44 ± 0.09
30 wt%	36 ± 12	75%	0.2 ± 0.1	0.25 ± 0.16
